# Preference for High Dietary Salt Intake Is Associated With Undiagnosed Type 2 Diabetes: The Henan Rural Cohort

**DOI:** 10.3389/fnut.2020.537049

**Published:** 2020-09-16

**Authors:** Tanko Abdulai, Tu Runqi, Zhenxing Mao, Timothy Bonney Oppong, Cecilia Amponsem-Boateng, Yan Wang, Xiaotian Liu, Haiqing Zhang, Chongjian Wang

**Affiliations:** ^1^Department of Epidemiology and Biostatistics, College of Public Health, Zhengzhou University, Zhengzhou, China; ^2^Department of Community Health and Family Medicine, School of Medicine and Health Sciences, University for Development Studies, Tamale, Ghana

**Keywords:** diabetes, undiagnosed diabetes, rural, obesity, dietary salt

## Abstract

**Background:** Diabetes continues to be a serious disease burden globally. Nutrition plays a vital role in human life and health, and both inadequate and over nutrition have been implicated in cause the of disease.

**Objective:** We explored the role of preference for high dietary salt intake and diabetes in a rural population. We also looked at possible mediating factors in the relationship between diabetes and preference for high dietary salt intake.

**Methods/Participants:** Data from the Henan rural cohort in China were analyzed. Using multinomial regression models, the association between diabetes diagnosis type and reported preference for dietary salt was assessed. Adjusted odds ratios (aORs) with confidence interval (CI) at a 95% level are reported. Mediation analyses using structural equation models in Stata were performed.

**Results:** Of the 39,183 participants included in the analyses, 4.07% were previously diagnosed with diabetes, and 5.80% with undiagnosed diabetes. Eighteen percent had a preference for salty meals, the mean age was 55.45 years, and 60% were women. Preference for salty meals was associated with undiagnosed diabetes (*aOR* = *1.17, 95% CI 1.03, 1.32*), but not with previously diagnosed diabetes. Stratified analysis revealed that the association with undiagnosed diabetes was only significant in men (*aOR* = *1.36, 95% CI 1.13, 1.70*) but not in women (*aOR* = *1.06, 95% CI 0.81, 1.38*). BMI and central obesity fully mediated the association. Dietary salt intake was self-reported and therefore presents a limitation.

**Conclusion:** Our study demonstrated that preference for high dietary salt intake is associated with undiagnosed diabetes but not prevalent diabetes.

## Introduction

Diabetes continues to be a serious disease burden in all countries. The risk factors and distribution has been extensively studied in the last half-century (1, 2). The risk factors for type 2 diabetes are broadly categorized into environmental and genetic causes. Of the environmental modifiable risk factors of type 2 diabetes, human nutrition plays a critical role in the development of the disease. The role of nutrients in the development of diabetes is linked to glucose metabolism, and this is as a result of the impairment of insulin regulation (3, 4). Red meat, processed meat, and sweetened beverages have been linked to increased risk of type diabetes (5).

Nutrition plays a vital role in human life and health, and both inadequate and over nutrition have been implicated in disease cause. Dietary salt is composed of sodium (Na) and chloride (Cl); Na is an essential element for the maintenance of fluid balance in humans (6), but higher intake of Na usually in the form of table salt is an established risk factor for cardiovascular diseases. Salt has been established to be associated with hypertension in many studies (7, 8), but the association between the intake of salt and diabetes has not received the same attention. The association of salt intake and diabetes has been suspected at least over the past five decades, when studies noted the susceptibility of desert rats to diabetes when they switched from the normal diet of desert vegetation (usually high in salt) to laboratory meals (9).

The World Health Organization (WHO) recommends a daily intake of sodium for adults to <87 mmol (<5 g of salt) and potassium intake to ≥90 mmol (≥3.5 g) per day (6, 10). In a recent meta-analysis and systemic review of salt consumption among Chinese in China, it was reported that China has the highest consumption of salt in the world nearly double the daily dietary recommended intake, the study further revealed that the Henan province and most of central china have the highest salt intake in the country (11).

This study explored the role of reported preference for high dietary salt intake and diabetes in a rural population. We also looked at possible mediating factors in the relationship between diabetes and preference for high dietary salt intake. Previous studies have demonstrated associations between high dietary salt intake and obesity (12–14) and since diabetes is strongly associated with obesity we are hypothesizing that any association between diabetes and dietary salt may be mediated by obesity.

## Methods

### Study Participants

This study is a population-based survey study, and men and women 18–79 years in five rural counties (Suiping, Tongxu, Xinxiang, Yima, and Yuzhou counties) of the Henan province in China were invited to participate. Invitations were sent to adults aged 18–79 in July 2015 and September 2017, 39,259 participants accepted the invitation to participate.

### Sampling and Data Collection

In brief, the Henan province was divided into five clusters based on geography (Northern, Southern, Central, Western and Eastern) and five counties (one from each geographic zone) randomly selected using a simple random sampling procedure. The local health authorities in the geographic zones then selected 1–3 local villages based on the estimated population requirement for each cluster. All permanent adult residents of the selected villages were then invited to participate in the study. The design of this study has been previously described elsewhere (15).

Trained staff administered questionnaires for sociodemographic information, lifestyle behaviors, history of diagnosed chronic diseases, family history of diabetes, and hypertension. Nurses and trained health personnel made anthropometric and blood pressure (BP) measurements, and collected venous fasting blood samples (after overnight fast). Study participants in light clothing with no shoes were weighed to the nearest 0.1 kg, using a digital scale. Height was determined using a standard tape measure to the nearest centimeter attached to a wall.

### Outcome Variable and Primary Exposure Measurement

Diabetes status was established primarily by measuring blood glucose level after an overnight fast; participants with glucose level ≥6.1 mmol/L but <7 mmol/L were classified as having impaired fasting glucose (IFG), those with glucose level ≥7 mmol/L with no prior diagnosis of diabetes were categorized as “undiagnosed diabetes,” and those who reported a previous diagnosis of diabetes by a physician were categorized as “diagnosed diabetes” (16).

Dietary salt intake was assessed by asking participants of the strength of their salty flavor preference (1 = light 2 = Moderate 3 = salty 4 = very salty). Responses indicating a preference for salty or very salty were considered as having a preference for high dietary salt and the responses light and moderate were coded as having no preference for high dietary salt.

### Other Covariates

Age, gender, education (categorized as; “less than high school,” “high school,” and “greater than high school”), marital (“married/cohabiting,” “widowed/divorced,” and “never married”), body mass index (BMI), dyslipidemia, hypertension, physical activity, smoking, and alcohol intake were the covariates adjusted for in the regression models. Dyslipidemia was defined using the IDF criteria for raised triglycerides, LDL-C, and reduced HDL-C or the use of lipid-lowering medications (2). Hypertension was considered if systolic blood pressure (SBP) measurement was >140 mmhg, diastolic blood pressure (DBS) >90 mmhg, or self-reported previous diagnosis of hypertension (17). BMI was categorized into three (normal weight, overweight and obese), and waist circumference (WC) into two (central obesity vs. no central obesity) using the WHO Asian population reference of BMI and WC for men and women (18).

### Laboratory Methods

All laboratory measurements for fasting glucose and blood lipids were performed by ROCHE Cobas C501 automatic biochemical analyzer by direct and indirect enzymatic methods (GOD-PAP, Switzerland).

### Data Analysis

Statistical analyses were performed using STATA 13 (Stata Corp., Tx). Summary statistics for participants are presented as counts and percentages for categorical variables and means with standard deviations (SD) for continuous variables. χ^2^ analysis was performed to assess the association between groups for categorical variables and student *t*-test used for continuous variables.

Diabetes status was first converted into a binary variable for those with diabetes (both diagnosed and undiagnosed diabetes) and those without diabetes (normoglycemia and impaired fasting glucose). Binary logistic regression models were then fitted to assess the associations of diabetes and risk factors. The goodness of fit test was conducted to assess the suitability of models.

Diabetes status was further classified into three categories of impaired fasting glucose, diagnosed diabetes and undiagnosed diabetes based on the WHO guidelines (16). Multinomial regression models were then performed to ascertain the associations of diabetes diagnosis status (IFG, previously diagnosed and undiagnosed diabetes) and the reported preference for high dietary salt intake. Age, gender, dyslipidemia, BMI, physical activity, family history of diabetes, and hypertension were adjusted for in a second model. All models were fitted using complex survey design approach in stata. Akaike information criterion (AIC) and Bayesian Information Criterion (BIC) were then used to assess model suitability.

Previous studies have reported association between obesity and high dietary salt intake; we, therefore, performed mediation analyses to test the hypothesis that the association between preference for salty meals and diabetes may be mediated through obesity. Mediation analyses were performed using structural equation models (medsem command in STATA). Candidate mediating variables were BMI and waist circumference (WC) and covariates considered were age, gender, physical activity level, family history of diabetes, and dyslipidemia. The mediation hypothesis was tested using the Baron and Kenny approach modified by Laobuti et al. and the Zhao et al. approach (19, 20).

## Results

In all, 39,183 participants from the Henan rural cohort were included in this present analysis, after excluding four participants with type 1 diabetes and 72 participants younger than 20 years. Over 60% were women, the mean age of participants was 55.67 ± 12.10 years. The overall prevalence of diabetes was 9.87% (5.80% were previously undiagnosed). Over 80% of the participants had lower than high school education, nearly 90% were married or in stable relationships, and close to 70% were from households that earned <1,000 RMB per month. Details of participants' characteristics are presented in Table 1.

**Table 1:** Baseline characteristics of participants stratified by diabetes diagnosis status.

		**Diabetes diagnosis status**				
	**Overall**	**Normoglycemic**	**IFG**	**UD**	**DD**	***P*-value**
	**39,183**	**32,397 (82.68)**	**2,918 (7.45)**	**2,273 (5.80)**	**1,595 (4.06)**	
**Gender**						0.072
Female	23,725 (60.55)	19,602 (82.62)	1,736 (7.32)	1,375 (5.80)	1,012 (4.27)	
Male	15,458 (39.45)	12,795 (82.77)	1,182 (7.65)	898 (5.81)	583 (3.77)	
**Education**						<0.001
< High school	33,203 (84.74)	27,359 (82.40)	2,513 (7.57)	1,916 (5.77)	1,415 (4.26)	
High school	4,852 (12.38)	4,039 (83.24)	366 (7.54)	292 (6.02)	155 (3.19)	
>High school	1,128 (2.88)	999 (88.56)	39 (3.46)	65 (5.76)	25 (2.22)	
**Marital Status**						<0.001
Married/cohabitating	35,230 (89.91)	29,207 (82.90)	2,570 (7.29)	2,034 (5.77)	1,419 (4.03)	
Widowed/divorce	3,406 (8.69)	2,699 (79.24)	323 (9.48)	215 (6.31)	169 (4.96)	
Single (never married)	547 (1.40)	491 (89.76)	25 (4.57)	24 (4.39)	7 (1.28)	
**BMI**						<0.001
Normal	16,646 (42.62)	14,693 (88.27)	851 (5.11)	637 (3.83)	465 (2.79)	
Overweight	15,462 (39.59)	12,515 (80.94)	1,270 (8.21)	987 (6.38)	690 (4.46)	
Obese	6,947 (17.79)	5,098 (73.38)	778 (11.20)	641 (9.23)	430 (6.19)	
**Central obesity**						<0.001
Yes	20,224 (51.72)	15,610 (77.19)	1,943 (9.61)	1,546 (7.64)	1,125 (5.56)	
No	18,877 (48.28)	16,727 (88.61)	964 (5.11)	723 (3.83)	463 (2.45)	
**Income (Yuan)**						<0.001
<1,000	26,884 (68.61)	22,082 (82.14)	2,091 (7.78)	1,558 (5.80)	1,153 (4.29)	
1,000~	9,385 (23.95)	7,832 (83.45)	647 (6.89)	549 (5.85)	357 (3.80)	
2,000~	1,915 (4.89)	1,628 (85.01)	117 (6.11)	109 (5.69)	61 (3.19)	
3,000~	999 (2.55)	855 (85.59)	63 (6.31)	57 (5.71)	24 (2.40)	
**Smoking**						<0.001
Never	28,512 (72.77)	23,502 (82.43)	2,106 (7.39)	1,683 (5.90)	1,221 (4.28)	
Ever (quit)	3,191 (8.14)	2,511 (78.69)	318 (9.97)	195 (6.11)	167 (5.23)	
current	7,480 (19.09)	6,384 (85.35)	494 (6.60)	395 (5.28)	207 (2.77)	
**Drinking**						<0.001
Never	30,287 (77.30)	25,059 (82.74)	2,209 (7.29)	1,741 (5.75)	1,278 (4.22)	
Ever (quit)	1,831 (4.67)	1,444 (78.86)	165 (9.01)	106 (5.79)	116 (6.34)	
Current	7,065 (18.03)	5,894 (83.43)	544 (7.70)	426 (6.03)	201 (2.85)	
**Vegetables/fruit intake**						<0.001
Yes	16,361 (41.76)	13,921 (85.09)	1,021 (6.24)	875 (5.35)	544 (3.32)	
No	22,820 (58.24)	18,475 (80.96)	1,897 (8.31)	1,397 (6.12)	1,051 (4.61)	
**High dietary Salt intake**						0.001
Yes	6,978 (17.83)	5,696 (81.63)	535 (7.67)	473 (6.78)	274 (3.93)	
No	32,154 (82.17)	26,663 (82.92)	2,375 (7.39)	1,798 (5.59)	1,318 (4.10)	
**Exercise**						<0.001
Low PA	12,676 (32.35)	10,096 (79.65)	1,092 (8.61)	818 (6.45)	670 (5.29)	
Moderate PA	14,784 (37.73)	12,405 (83.91)	1,003 (6.78)	828 (5.60)	548 (3.71)	
Vigorous/intense PA	11,723 (29.92)	9,896 (84.42)	823 (7.02)	627 (5.35)	377 (3.22)	
**Hypertensive**						<0.001
Yes	12,836 (32.79)	9,551 (74.41)	1,438 (11.20)	1,014 (7.90)	833 (6.49)	
No	26,312 (67.21)	22,818 (86.72)	1,476 (5.61)	1,256 (4.77)	762 (2.90)	
**Family history of diabetes**						<0.001
Yes	1,645 (4.20)	745 (45.29)	88 (5.35)	558 (33.92)	254 (15.44)	
No	37,538 (95.80)	31,652 (84.32)	2,830 (7.54)	1,715 (4.57)	1,341 (3.57)	
Age, mean ± SD	55.67 ± 12.10	54.93 ± 12.41	59.31 ± 10.29	56.50 ± 11.31	61.20 ± 8.72	<0.001
BMI, mean ± SD	24.84 ± 3.57	24.57 ± 3.49	26.01 ± 3.64	26.23 ± 3.72	26.01 ± 3.65	<0.001
WC, mean ± SD	84.10 ± 10.40	83.15 ± 10.17	88.17 ± 10.40	88.76 ± 10.48	88.99 ± 10.15	0.095
TC, mean ± SD	4.76 ± 0.99	4.70 ± 0.95	5.06 ± 1.12	5.06 ± 1.13	5.02 ± 1.12	<0.001
TG, mean ± SD	1.68 ± 1.12	1.61 ± 1.05	1.91 ± 1.28	2.08 ± 1.40	2.13 ± 1.47	<0.001
HDLC, mean ± SD	1.32 ± 0.33	1.34 ± 0.33	1.28 ± 0.34	1.24 ± 0.33	1.23 ± 0.32	<0.001
LDLC, mean ± SD	2.87 ± 0.82	2.83 ± 0.80	3.04 ± 0.91	3.03 ± 0.92	3.03 ± 0.96	<0.001

*Counts and percentages (in parenthesis) are presented unless otherwise stated*.

*BMI, Body mass index; WC, Waist circumference; TC, Total cholesterol; TG, Triglycerides HDLC=High density lipoprotein cholesterol; LDLC, Low density lipoprotein IFG, impaired fasting glucose; DD, Diagnosed diabetes; UD, Undiagnosed diabetes; SD, standard deviation*.

The reported prevalence of preference for high dietary salt intake was 18%, men, had a higher preference for salty meals compared to women. Additionally, participants with higher education, those who were overweight/obese, from households with high income, and currently drinking also reported high salty meals preference (Table 2).

**Table 2:** Baseline characteristics stratified by reported salt preference.

	**Preference for high dietary Salt intake**		
	**Yes**	**No**	***P*-value**
	**6,978 (17.83)**	**32,154 (82.17)**	
**Gender**			<0.001
Female	3,907 (16.49)	19,787 (83.51)	
Male	3,071 (19.89)	12,367 (80.11)	
**Education**			0.011
< High school	5,839 (17.60)	27,331 (82.40)	
High school	912 (18.85)	3,927 (81.15)	
>High school	227 (20.21)	896 (79.79)	
**Marital Status**			0.003
Married/cohabitating	6,332 (17.99)	28,858 (82.01)	
Widowed/divorce	537 (15.80)	2,861 (84.20)	
Single (never married)	109 (20.04)	435 (79.96)	
**BMI**			<0.001
Normal weight	2,687 (16.16)	13,936 (83.84)	
Overweight	2,819 (18.25)	12,624 (81.75)	
Obese	1,452 (20.93)	5,486 (79.07)	
**Central obesity**			<0.001
Yes	3,796 (18.79)	16,402 (81.21)	
No	3,171 (16.82)	15,682 (83.18)	
**Income (Yuan)**			<0.001
<1,000	4,538 (16.90)	22,308 (83.10)	
1,000~	1,865 ((19.89)	7,512 (80.11)	
2,000~	371 (19.40)	1,541 (80.60)	
3,000~	204 (20.46)	793 (79.54)	
**Smoking status**			<0.001
Never	4,718 (16.57)	23,755 (83.43)	
Ever (quit)	611 (19.17)	2,576 (80.83)	
current	1,649 (22.07)	5,823 (77.93)	
**Drinking status**			<0.001
Never	4,965 (16.42)	25,278 (83.58)	
Ever (quit)	330 (18.05)	1,498 (81.95)	
current	1,683 (23.84)	5,378 (76.16)	
**Vegetables/fruits intake**			0.038
yes	2,839 (17.36)	13,516 (82.64)	
no	4,139 (18.17)	18,638 (81.83)	
**Exercise**			<0.001
Low Physical Activity	2,140 (16.91)	10,516 (83.09)	
Moderate Physical Activity	2,495 (16.89)	12,275 (83.11)	
Vigorous/intense Physical Activity	2,343 (20.02)	9,363 (79.98)	
**Hypertension**			0.267
Yes	2,325 (18.14)	10,492 (81.86)	
No	4,647 (17.68)	21,634 (82.32)	
**Diabetes**			0.001
Normoglycemic	5,696 (17.60)	26,663 (82.40)	
Impaired fasting glucose	535 (18.38)	2,375 (81.62)	
Undiagnosed diabetes	473 (20.83)	1,798 (79.17)	
Diagnosed diabetes	274 (17.21)	1,318 (82.79)	
**Family history of diabetes**			<0.001
Yes	347 (21.12)	1,296 (78.88)	
No	6,631 (17.69)	30,858 (82.31)	
Age, mean ± SD	54.46 ± 12.22	55.85 ± 12.16	0.592
BMI, mean ± SD	25.18 ± 3.64	24.76 ± 3.55	0.003
WC, mean ± SD	85.24 ± 10.54	83.83 ± 10.36	0.064
TC, mean ± SD	4.75 ± 4.75	4.76 ± 0.99	0.168
TG, mean ± SD	1.68 ± 1.14	1.68 ± 1.12	0.089
HDLC, mean ± SD	1.30 ± 0.33	1.33 ± 0.34	0.024
LDLC, mean ± SD	2.89 ± 0.83	2.86 ± 0.82	0.126

*Counts and percentages (in parenthesis) are presented unless otherwise stated*.

*BMI, Body mass index; WC, Waist circumference; TC, Total cholesterol; TG, Triglycerides; HDLC, High density lipoprotein cholesterol; LDLC, Low density lipoprotein; SD, standard deviation*.

Diabetes presence was associated with the traditional risk factors as reported in previous studies; family history (*aOR* = *3.46, 95% CI 2.77, 4.32)*, dyslipidemia (*aOR* = *1.54, 95% CI 1.30, 1.83*), waist circumference (*aOR* = *1.68, 95% CI 1.42, 1.98*), BMI (*aOR* = *1.25, 95% CI 1.14, 1.38*), age (*aOR* = *1.04, 95% CI 1.03, 1.05*), level of physical activity (*aOR* = *0.86, 95% CI 0.80, 0.92*), and gender (*aOR* = *0.73, 95% CI 0.61, 0.88*). Men were at increased odds of having diabetes compared to women; similarly, older participants were also at increased odds of diabetes (Supplementary Table 1).

### Salt and Diabetes

Combined prevalent diabetes and newly diagnosed diabetes was not associated with a reported preference for salty meals in binary logistic regression models (*aOR* = *1.02, 95% CI 0.88, 1.20*). However, in multinomial regression analysis, it was found to be significant among participants with undiagnosed (*aOR* = *1.17, 95% CI 1.03, 1.32*) and further stratified analysis revealed the association was only significant among men but not women (men: *aOR* = *1.36, 95% CI 1.13, 1.70;* women*: aOR* = *1.06, 95% CI 0.81, 1.38*). The results are presented in Tables 3, 4. Association of diabetes and covariates is shown in Supplementary Tables 2, 3.

**Table 3:** Binary logistic regression, association of salt and diabetes (both diagnosed and undiagnosed).

**Diabetes**	**Unadjusted model**		Adjusted model^*^	
	**OR (95% CI)**	***P*-value**	**OR (95% CI)**	***P*-value**
All	1.01 (0.86, 1.15)	0.953	1.02 (0.88, 1.20)	0.768
Men	1.10 (0.87, 1.38)	0.404	1.11 (0.88, 1.40)	0.393
Women	0.95 (0.78, 1.16)	0.611	0.99 (0.80, 1.21)	0.904

* *Adjusted for age, gender, BMI, hypertension, family history of diabetes, and physical activity*.

**Table 4:** Multinomial regression, association of salt and diabetes diagnosis status.

**Diabetes diagnosis type**	**Unadjusted model**		Adjusted model^*^	
	**All Participants**			
	**RRR (95% CI)**	***P*****-value**	**RRR (95% CI)**	***P*****-value**
Impaired fasting glucose	0.87 (0.73,1.03)	0.096	0.87 (0.73, 1.03)	0.107
Undiagnosed Diabetes	**1.22 (1.08, 1.40)**	** <0.030**	**1.17 (1.03, 1.32)**	**0.021**
Diagnosed Diabetes	0.96 (0.77, 1.22)	0.730	1.00 (0.79, 1.26)	0.986
	**Men**			
Impaired fasting glucose	0.91 (0.71, 1.17)	0.473	0.90 (0.70, 1.15)	0.389
Undiagnosed Diabetes	**1.46 (1.12, 1.91)**	** <0.006**	**1.36 (1.13, 1.70)**	** <0.028**
Diagnosed Diabetes	1.04 (0.73, 1.48)	0.838	1.07 (0.75, 1.55)	0.700
	**Women**			
Impaired fasting glucose	0.83 (0.66, 1.00)	0.119	0.84 (0.66, 1.07)	0.151
Undiagnosed Diabetes	1.10 (0.86, 1.41)	0.438	1.06 (0.81, 1.38)	0.671
Diagnosed Diabetes	0.92 (0.69, 1.23)	0.589	0.96 (0.71, 1.31)	0.808

*RRR, Relative risk ratio*.

* *Adjusted for age, gender (only in all participants), BMI, hypertension, family history of diabetes, and physical activity*.

*Bold value means statistically significant*.

The association between undiagnosed diabetes and reported a preference for salty meals was fully mediated by BMI and waist circumference using both the Baron and Kenny approach and Zhao et al. method (see Supplementary Tables 4, 5).

## Discussion

Type 2 diabetes continues to be a significant global disease burden and the full understanding of the causes is still evolving. About one in ten of the participants at baseline in the Henan rural cohort had type 2 diabetes. Persons with type 2 diabetes had the classic risk factors reported in previous studies (2, 3); they were overweight/obese, more centrally obese, dyslipidemic, hypertensive, men, more likely to have had a family history of diabetes, and older compared to those without diabetes. There were a fairly equal number of prevalent type 2 diabetes and undiagnosed diabetes.

Preference for high dietary salt was positively associated with undiagnosed diabetes but not prevalent diabetes. The lack of association with prevalent diabetes may be attributable to the modification of lifestyle behavior following counsel from physicians/dieticians/nutritionists as patients with diagnosed diabetes are usually advised to limit dietary salt intake to lower their risk for cardiovascular diseases (21). In stratified analysis by gender, this association was only significant among men but not women. From our study population with undiagnosed diabetes, men had a 50% more preference for high dietary salt compared to women; this may partly explain the gender difference in associations of undiagnosed diabetes and preference for high dietary salt. Men and women generally have different lifestyles, with women generally adopting more healthy lifestyles compared to men. In recent studies of dietary behaviors with respect to salt intake by Briar et al. and Gray et al., women were more likely to report and have less salt intake, and eat less from outside compared to men (22, 23).

The mechanism of the association between diabetes and salt is not yet fully understood, but previous animal studies suggest that high salt intake is associated with increased fatty acid absorption (13). Observational population studies have also indicated that high salt intake is positively associated with obesity (12–14). The mechanism for the association of obesity and high salt intake has been proposed as a result of the increased thirst and satiation created by high salt intake and demand for more fluids and energy intake (24). Recent studies in both humans and mice, however, suggest that high salt intake does not create more thirst, as it actually lessens the requirement of less fluid intake by increased urea synthesis which causes more reabsorption of water. The increased urea synthesis is a high energy demand activity and thus causes satiation making subjects with high salt intake more hungrier (25, 26).

Persons with prevalent diabetes knew their diabetes status and had encountered physicians or other health professionals with regards to the management of their condition; as part of the management of diabetes condition, patients are counseled to avoid lifestyles that will predispose them to cardiovascular diseases and limiting salt intake is one of the key management strategies of hypertension and subsequent cardiovascular diseases (27). In a prospective study of dietary salt intake and diabetes, salt was found to be associated with incident diabetes (28).

This study is perhaps the first to explore the explanatory reason for the association of high dietary salt intake and diabetes. We conducted a mediation analysis and gleaning from the literature there are previous studies that established an association between high dietary intake and obesity (12, 14, 24). We, therefore, considered BMI and WC as candidate mediating factors for the mediation analyses. Both BMI and WC, and salt intake were highly correlated within our study population. BMI and WC fully mediated the association between preference for high dietary salt intake and diabetes. This offers an important explanation of the association of dietary salt and diabetes as reported in previous studies (28, 29).

These findings represent yet another important dietary determinant of type 2 diabetes. One strength of the present study is the large sample size and the rigorous control of multiple covariates. Some limitations are, however, inevitable; diabetes status was determined using fasting blood glucose, we could have, therefore, missed some cases. In this study, the quantity and frequency of salt intake were not assessed; we were also unable to use objective measurements such as 24 h urinary Sodium measurement alongside the self-reported salt flavor preference. Relying on self-report may inevitably introduce reporting bias. But a previous study demonstrated a good correlation of self-reported dietary salt intake and a 24 h urine assay of salt (30). This study, being cross sectional by design cannot establish a causal relationship between dietary salt and diabetes.

Energy intake was not assessed and, therefore, not controlled for in our adjusted models. The effect of residual confounding due to measurement error in the assessment of confounding factors is also inevitable.

## Conclusion

Our study demonstrated that preference for high intake of dietary salt is associated with undiagnosed diabetes but not prevalent diabetes. This association is fully mediated by obesity. More effort should, therefore, be directed toward limiting salt intake through education and strict regulation of processed foods. More prospective studies are needed to validate our findings.

## Data Availability Statement

All datasets generated for this study are included in the article/Supplementary Material.

## Ethics Statement

The studies involving human participants were reviewed and approved by the ethics committee of the Zhengzhou University live sciences ethics committee. All participants signed informed consent forms before the commencement of the study. Ethics approval code: [2015] MEC (S128). The patients/participants provided their written informed consent to participate in this study.

## Author Contributions

CW conceived, designed the study, and secured funding. TA, TO, and CA-B analyzed the data. TA wrote the first draft of the manuscript. TR, XL, YW, and HZ supervised the data collection. ZM provided technical direction and writing assistance in the preparation of this manuscript. All authors critically revised the manuscript and approved the final version to be published.

## Conflict of Interest

The authors declare that the research was conducted in the absence of any commercial or financial relationships that could be construed as a potential conflict of interest.
