# Supplementary material for: Preference for High Dietary Salt Intake Is Associated With Undiagnosed Type 2 Diabetes: The Henan Rural Cohort
Source: Front Nutr. 2020 Sep 16;7:537049. doi: 10.3389/fnut.2020.537049 (PMC7557361; doi:10.3389/fnut.2020.537049)
Supplement: Supplementary file 1 [file Data_Sheet_1.docx]

**Supplementary tables**

**Title: Preference for high dietary salt intake is associated with undiagnosed type 2 diabetes: the Henan Rural Cohort**

Table S1: Binary logistic regression; association of salt (with covariates) and diabetes (both diagnosed and undiagnosed)

| Combined diagnosed and undiagnosed | Unadjusted model | | Adjusted model* | |
| --- | --- | --- | --- | --- |
| All participants | OR(95% CI) | P-value | OR(95% CI) | P-value |
| Salt intake | 1.01(0.86, 1.15) | 0.953 | 1.02(0.88, 1.20) | 0.768 |
| Age | 1.04(1.03, 1.05) | <0.001 | **1.04(1.03, 1.05)** | **<0.001** |
| Gender | 1.03(0.92, 1.16) | 0.568 | **0.73(0.61, 0.88)** | **0.001** |
| Smoking | 0.90(0.83, 0.97) | <0.005 | 0.91(0.80, 1.02) | 0.107 |
| Drinking | 0.89(0.82, 0.95) | 0.001 | 0.91(0.82, 1.00) | 0.057 |
| Exercise | 0.78(0.73, 0.84) | <0.001 | **0.86(0.80, 0.92)** | **<0.001** |
| Vegetables/fruits intake | 0.71(0.62, 0.80) | <0.001 | **0.79(0.69, 0.90)** | **0.001** |
| Hypertension | 2.08(1.86, 2.33) | <0.001 | **1.42(1.26, 1.62)** | **<0.001** |
| Family history of diabetes | 2.45(2.01, 2.99) | <0.001 | **3.46(2.77, 4.32)** | **<0.001** |
| Waist circumference | 2.23(1.96, 2.53) | <0.001 | **1.68(1.42, 1.98)** | **<0.001** |
| Dyslipidemia | 2.02(1.71, 2.38) | <0.001 | **1.54(1.30, 1.83)** | **<0.001** |
| Education | 0.71(0.61, 0.83) | <0.001 | 0.89(0.75, 1.06) | 0.193 |
| BMI | 1.59(1.48, 1.71) | <0.001 | **1.25(1.14, 1.38)** | **<0.001** |
|  |  |  |  |  |
| Men |  |  |  |  |
| Salt intake | 1.10(0.87, 1.38) | 0.404 | 1.11(0.88, 1.40) | 0.393 |
| Age | 1.02(1.01, 1.03) | <0.001 | **1.03(1.02, 1.04)** | **<0.001** |
| Smoking | 0.81(0.73, 0.89) | <0.001 | **0.87(0.78, 0.99)** | **0.029** |
| Drinking | 0.87(0.79, 0.96) | 0.005 | 0.90(0.81, 1.00) | 0.060 |
| Exercise | 0.74(0.67, 0.83) | <0.001 | **0.81(0.72, 0.90)** | **<0.001** |
| Vegetables/fruits intake | 0.95(0.77, 1.16) | <0.597 | 1.04(0.84, 1.28) | 0.745 |
| Hypertension | 1.74(1.44, 2.10) | <0.001 | **1.27(1.03, 1.56)** | **0.026** |
| Family history of diabetes | 2.88(2.14, 3.89) | <0.001 | **3.59(2.55, 5.06)** | **<0.001** |
| Waist circumference | 2.26(1.87, 2.73) | <0.001 | **1.54(1.16, 2.05)** | **0.003** |
| Dyslipidemia | 1.95(1.51, 2.52) | <0.001 | **1.66(1.27, 2.17)** | **<0.001** |
| Education | 0.94(0.79, 1.12) | 0.499 | 0.99(0.81, 1.20) | 0.928 |
| BMI | 1.70(1.51, 1. 91) | <0.001 | **1.33(1.11, 1.60)** | **0.002** |
| Women |  |  |  |  |
| Salt intake | 0.95(0.78, 1.16) | 0.611 | 0.99(0.80, 1.21) | 0.904 |
| Age | 1.05(1.04, 1.06) | <0.001 | **1.05(1.04, 1.06)** | **<0.001** |
| Smoking | 1.69(1.12, 2.56) | 0.013 | 1.58(0.99, 2.51) | 0.053 |
| Drinking | 0.74(0.57, 0.97) | <0.026 | 0.82(0.62, 1.06) | 0.138 |
| Exercise | 0.82(0.74, 0.89) | <0.001 | **0.88(0.81, 0.97)** | **0.012** |
| Vegetables/fruits intake | 0.59(0.50, 0.70) | <0.001 | **0.67(0.57, 0.97)** | **<0.001** |
| Hypertension | 2.31(1.99, 2.67) | <0.001 | **1.50(1.27, 1.75)** | **<0.001** |
| Family history of diabetes | 2.27(1.76, 2.95) | <0.001 | **3.61(2.71, 4.80)** | **<0.001** |
| Waist circumference | 2.48(2.07, 2.97) | <0.001 | **1.70(1.39, 2.09)** | **<0.001** |
| Dyslipidemia | 2.06(1.66, 2.55) | <0.001 | **1.42(1.14, 1.77)** | **0.002** |
| Education | 0.53(0.40, 0.70) | <0.001 | 0.75(0.55, 1.02) | 0.064 |
| BMI | 1.54(1.40, 1.69) | <0.001 | **1.21(1.09, 1.36)** | **0.001** |

OR= Odds ratio, BMI= Body mass index

* Adjusted for age, gender (only in all participants), BMI, hypertension, family history of diabetes, and physical activity

Table S2: Multinomial regression; association of salt (with covariates) and diabetes diagnosis status

|  | Unadjusted model | | Adjusted model* | |
| --- | --- | --- | --- | --- |
| Impaired Fasting Glucose | RRR(95% CI) | P-value | RRR(95% CI) | P-value |
| Salt intake | 0.87(0.73,1.03) | 0.096 | 0.87(0.73, 1.03) | 0.107 |
| Age | 1.03(1.02, 1.04) | <0.001 | **1.03(1.02, 1.03)** | **<0.001** |
| Gender | 0.95(0.83, 1.08) | 0.407 | **0.87 (0.72, 1.05)** | **<0.001** |
| Smoking | 0.98(0.90, 1.05) | 0.537 | 0.95(0.85, 1.06) | 0.399 |
| Drinking | 1.04(0.96, 1.12) | 0.360 | 1.07(0.97, 1.18) | 0.207 |
| Exercise | 0.90(0.83, 0.97) | 0.009 | 0.97(0.90, 1.05) | 0.463 |
| Vegetables | 0.70(0.61, 0.81) | <0.001 | **0.78(0.67, 0.91)** | **0.001** |
| Hypertension | 2.25(1.98, 2.56) | <0.001 | **1.65(1.44, 1.90)** | **<0.001** |
| Waist circumference | 1.85(1.61, 2.12) | <0.001 | **1.40(1.16, 1. 70)** | **<0.001** |
| Dyslipidemia | 1.85(1.56, 2.20) | <0.001 | **1.42(1.19, 1.70)** | **<0.001** |
| Education | 0.94(0.79, 1.12) | 0.505 | 1.18(0.97, 1.44) | 0.091 |
| Family history of diabetes | 1.41(0.99, 2.03) | 0.060 | **1.96(1.34, 2.88)** | **<0.001** |
| Marital | 1.23(1.05, 1.45) | 0.012 | 1.06(0.89, 1.26) | 0.534 |
| BMI | 1.52(1.39, 1.65) | <0.001 | **1.26(1.12, 1.42)** | **<0.001** |
| Undiagnosed Diabetes |  |  |  |  |
| Salt intake | **1.22(1.08, 1.40)** | **<0.030** | **1.17(1.03, 1.32)** | **0.021** |
| Age | 1.01(1.00, 1.02) | 0.094 | **1.02(1.01, 1.02)** | **<0.001** |
| Gender | 0.96(0.83, 1.12) | 0.597 | **0.74(0.58, 0.93)** | **0.011** |
| Smoking | 0.99(0.90, 1.09) | 0.868 | 0.98(0.84, 1.14) | 0.757 |
| Drinking | 1.02(0.94, 1.12) | 0.615 | 0.97(0.86, 1.11) | 0.684 |
| Exercise | 0.85(0.78, 0.93) | <0.001 | **0.88(0.80, 0.96)** | **0.006** |
| Vegetables/fruits intake | 0.82(0.70, 0.96) | 0.012 | **0.84(0.71, 0.99)** | **0.038** |
| Hypertension | 1.53(1.32, 1.78) | <0.001 | **1.30(1.10, 1.53)** | **0.002** |
| Waist circumference | 2.04(1.74, 2.38) | <0.001 | **1.54(1.24, 1.91)** | **<0.001** |
| Dyslipidemia | 1.82(1.49, 2.23) | <0.001 | **1.56(1.28, 1.92)** | **<0.001** |
| Education | 1.14(0.96, 1.35) | 0.132 | 1.09(0.91, 129) | 0.355 |
| Family history of diabetes | 11.99(9.77, 14.71) | <0.001 | **14.67(11.75, 18.32)** | **<0.001** |
| Marital | 1.08(0.89, 1.32) | 0.417 | 1.14(0.91, 1.42) | 0.255 |
| BMI | 1.63(1.49, 1.79) | <0.001 | **1.28(1.13, 1.46)** | **<0.001** |
| Diagnosed diabetes |  |  |  |  |
| Salt intake | 0.96(0.77, 1.22) | 0.730 | 1.00(0.79, 1.26) | 0.986 |
| Age | 1.05(1.04, 1.06) | <0.001 | **1.06(1.05, 1.07)** | **<0.001** |
| Gender | 1.11(0.93, 1.32) | 0.253 | **0.68(0.51, 0.91)** | **0.009** |
| Smoking | 0.82(0.73, 0.91) | <0.001 | **0.80(0.67, 0.95)** | **0.013** |
| Drinking | 0.85(0.76, 0.95) | 0.004 | 0.93(0.81, 1.07) | 0.328 |
| Exercise | 0.74(0.67, 0.82) | <0.001 | **0.82(0.73, 0.91)** | **<0.001** |
| Vegetables/fruits intake | 0.68(0.56, 0.83) | <0.001 | **0.77(0.63, 0.94)** | **0.011** |
| Hypertension | 2.36(2.00, 2.80) | <0.001 | **1.56(1.30, 1.89)** | **<0.001** |
| Waist circumference | 2.41(2.01, 2.89) | <0.001 | **1.82(1.43, 2.32)** | **<0.001** |
| Dyslipidemia | 2.22(1.72, 2.87) | <0.001 | **1.73(1.32, 2.25)** | **<0.001** |
| Education | 0.65(0.53, 0.80) | <0.001 | 0.83(0.65, 1.05) | 0.119 |
| Family history of diabetes | 6.30(4.86, 8.18) | <0.001 | **11.52 (8.53, 15.55)** | **<0.001** |
| Marital | 1.11(0.88, 1.39) | 0.393 | 0.82 (0.61, 1.08) | 0.155 |
| BMI | 1.61(1.44, 1.80) | <0.001 | **1.23 1.07, 1.41)** | **0.003** |

RRR= Relative risk ratio

* Adjusted for age, gender, BMI, hypertension, family history of diabetes, and physical activity

Table S3: Multinomial regression; association of salt (with covariates) and diabetes diagnosis status, stratified by gender

|  | Male | | | | Female | | | |
| --- | --- | --- | --- | --- | --- | --- | --- | --- |
|  | **Unadjusted model** | | **Adjusted model*** | | **Unadjusted model** | | **Adjusted model*** | |
| Impaired Fasting Glucose | **RRR(95% CI)** | **P-value** | **RRR(95% CI)** | **P-value** | **RRR(95% CI)** | **P-value** | **RRR(95% CI)** | **P-value** |
| Salt intake | 0.91(0.71, 1.17) | 0.473 | 0.90(0.70, 1.15) | 0.389 | 0.83(0.66, 1.00) | 0.119 | 0.84(0.66, 1.07) | 0.151 |
| Age | 1.01(1.00, 1.02) | 0.012 | **1.02(1.01¸ 1.03)** | **0.002** | 1.05(1.04, 1.06) | <0.001 | **1.04(1.03, 1.05)** | **<0.001** |
| Smoking | 0.90(0.81, 0.99) | 0.033 | 0.92(0.83, 1.03) | 0.146 | 1.31(0.86, 2.00) | 0.207 | 1.21 (0.78, 1.87) | 0.3947 |
| Drinking | 1.10(0.99, 1.22) | 0.069 | 1.10(0.98, 1.23) | 0.112 | 0.62(0.48, 0.79) | <0.001 | **0.69(0.53, 0.88)** | **0.003** |
| Exercise | 0.96(0.86, 1.07) | 0.465 | 1.03(0.91, 1.15) | 0.679 | 0.85(0.76, 0.95) | 0.005 | 0.92(0.83, 1.03) | 0.142 |
| Vegetables/fruits intake | 0.70(0.57, 0.86) | 0.001 | **0.75 (0.61, 0.93)** | **0.008** | 0.71(0.58, 0.86) | <0.001 | **0.81(0.66, 0.99)** | **0.036** |
| Hypertension | 1.93(1.58, 2.35) | <0.001 | **1.56(1.27, 1.92)** | **<0.001** | 2.48(2.09, 2.93) | <0.001 | **1.67(1.38, 2.01)** | **<0.001** |
| Waist circumference | 1.93(1.58, 2.36) | <0.001 | 1.33 (0.99, 1.78) | 0.058 | 2.06(1.67, 2.53) | <0.001 | **1.40(1.08, 1.80)** | **0.010** |
| Dyslipidemia | 1.39(1.06, 1.81) | 0.016 | 1.11 (0.84, 1.46) | 0.455 | 2.26(1.80, 2.84) | <0.001 | **1.61(1.27, 2.04** | **<0.001** |
| Education | 1.05(0.84, 1.32) | 0.640 | 1.07 (0.85, 1.36) | 0.551 | 0.84(0.64, 1.11) | 0.221 | 1.29(0.95, 1.76) | 0.103 |
| Family history of diabetes | 2.16(1.14, 4.10) | 0.018 | **2.47 (1.24, 4.92)** | **0.010** | 1.15(0.76, 1.75) | 0.503 | **1.80(1.16, 2.80)** | **0.009** |
| Marital | 0.80(0.61, 1.03) | 0.086 | 0.82 (0.62, 1.07) | 0.144 | 1.60(1.29, 1.99) | <0.001 | 1.15(0.91, 1.45) | 0.241 |
| BMI | 1.62(1.43, 1.83) | <0.001 | **1.35(1.11, 1.64)** | **0.003** | 1.47(1.31, 1.65) | <0.001 | **1.23(1.06, 1.42)** | **0.005** |
|  | | | | | | | | |
| Undiagnosed Diabetes |  |  |  |  |  |  |  |  |
| Salt intake | **1.46(1.12, 1.91)** | **0.006** | **1.36(1.13, 1.70)** | **0.028** | 1.10(0.86, 1.41) | 0.438 | 1.06(0.81, 1.38) | 0. 671 |
| Age | 0.99(0.98, 1.00) | 0.066 | **1.01(1.00, 1.02)** | **0.027** | 1.02(1.01, 1.02) | <0.001 | **1.02(1.01, 1.03)** | **<0.001** |
| Smoking | 0.93(0.81, 1.06) | 0.279 | 0.96(0.82, 1.12) | 0.607 | 1.68(0.97, 2.92) | 0.062 | 1.72(0.92, 3.19) | 0.088 |
| Drinking | 0.98(0.87, 1.11) | 0.774 | 0.92(0.79, 1.06) | 0.230 | 1.14(0.91, 1.44) | 0.247 | 1.08(0.87, 1.36) | 0.478 |
| Exercise | 0.87(0.75, 1.00) | 0.058 | 0.89 (0.77, 1.04) | 0.149 | 0.83(0.75, 0.93) | 0.001 | **0.85 (0.76, 0.96)** | **0.008** |
| Vegetables/fruits intake | 0.97(0.75, 1.25) | 0.799 | 1.00 (0.76, 1.32) | 0.976 | 0.73(0.60, 0.90) | 0.002 | 0.75 (0.61, 0.92) | 0.006 |
| Hypertension | 1.47(1.16, 1.86) | 0.002 | 1.21 (0.92, 1.57) | 0.167 | 1.57(1.30, 1.90) | <0.001 | **1.35 (1.10, 1.67)** | **0.005** |
| Waist circumference | 2.35(1.85, 2.98) | <0.001 | 1.38 (0.97, 1.96) | 0.075 | 2.10(1.70, 2.60) | <0.001 | **1.56 (1.20, 2.03)** | **0.001** |
| Dyslipidemia | 1.76(1.25, 2.48) | 0.001 | **1.51 (1.09, 2.10)** | **0.014** | 1.86(1.45, 2.38) | <0.001 | **1.56 (1.20, 2.01)** | **0.001** |
| Education | 1.20(0.97, 1.48) | 0.091 | 1.09(0.86, 1.37) | 0.474 | 1.08(0.83, 1.41) | 0.543 | 1.07 (0.82, 1.39) | 0.632 |
| Family history of diabetes | 14.40(10.38, 19.96) | <0.001 | **16.66 (11.70, 23.72)** | **<0.001** | 11.28 (8.72, 14.57) | <0.001 | **14.33 (10.92, 18.81)** | **<0.001** |
| Marital | 0.77 (0.60, 0.97) | 0.030 | 0.81 (0.62, 1.06) | 0.125 | 1.34(1.02, 1.76) | 0.034 | 1.34 (0.98, 1.85) | 0.070 |
| BMI | 1.87(1.61, 2.18) | <0.001 | **1.51 (1.20, 1.91)** | **<0.001** | 1.51(1.34, 1.70) | <0.001 | **1.19(1.03, 1.39)** | **0.022** |
|  | | | | | | | | |
| Diagnosed diabetes |  |  |  |  |  |  |  |  |
| Salt intake | 1.04(0.73, 1.48) | 0.838 | 1.07(0.75, 1.55) | 0.700 | 0.92(0.69, 1.23) | 0.589 | 0.96(0.71, 1.31) | 0.808 |
| Age | 1.03(1.02, 1.05) | <0.001 | **1.05(1.03, 1.06)** | **<0.001** | 1.06(1.05, 1.07) | <0.001 | **1.06(1.05, 1.07)** | **<0.001** |
| Smoking | 0.73(0.62, 0.84) | <0.001 | **0.77 (0.65, 0.92)** | **0.004** | 1.43(0.69, 2.95) | 0.332 | 1.34 (0.64, 2.81) | 0.435 |
| Drinking | 0.85(0.74, 0.97) | 0.019 | 0.93(0.80, 1.08) | 0.331 | 0.77(0.49, 1.21) | 0.257 | 0.83 (0.52,1.34) | 0.455 |
| Exercise | 0.68(0.58, 0.79) | <0.001 | **0.75 (0.64, 0.88)** | **<0.001** | 0.78(0.68, 0.91) | 0.001 | **0.86 (0.74, 0.99)** | **0.032** |
| Vegetables/fruits intake | 0.86(0.63, 1.17) | 0.337 | 0.97 (0.70, 1.34) | 0.862 | 0.60(0.46, 0.77) | <0.001 | **0.67(0.52, 0.87)** | **0.003** |
| Hypertension | 1.85(1.40, 2.45) | <0.001 | 1.27 (0.93, 1.74) | 0.135 | 2.70 (2.18, 3.34) | <0.001 | **1.72(1.36, 2.17)** | **<0.001** |
| Waist circumference | 2.69(2.03, 3.56) | <0.001 | **2.26 (1.48¸ 3.45)** | **<0.001** | 2.41(1.86, 3.13) | <0.001 | **1.55(1.16 2.07)** | **0.003** |
| Dyslipidemia | 2.21(1.49, 3.26) | <0.001 | **1.98 ( 1.34, 2.92)** | **0.001** | 2.24(1.61, 3.11) | <0.001 | **1.57(1.11 2.21)** | **0.010** |
| Education | 0.92(0.71, 1.18) | 0.495 | 1.00 (0.74, 1.35) | 0.993 | 0.46(0.32, 0.65) | <0.001 | **0.63(0.42, 0.94)** | **0.025** |
| Family history of diabetes | 7.86(5.30, 11.66) | <0.001 | **13.16 (8.20, 21.14)** | **<0.001** | 5.74(4.13, 7.98) | <0.001 | **11.69(7.98, 17.12)** | **<0.001** |
| Marital | 0.69 (0.46, 1.03) | 0.069 | 0.61(0.38, 0.98) | 0.043 | 1.40(1.04, 1.88) | 0.026 | 0.89(0.62, 1.28) | 0.539 |
| BMI | 1.67(1.42, 1.96) | <0.001 | 1.12 (0.86, 1.46) | 0.404 | 1.57(1.36, 1.81) | <0.001 | 1.27(1.08, 1.49) | 0.004 |

RRR= Relative risk ratio

* Adjusted for age, BMI, hypertension, family history of diabetes, and physical activity

Table S4: Mediation analysis of BMI on the salt 🡺 diabetes association

| Estimates | Delta | Sobel | Monte Carlo |
| --- | --- | --- | --- |
| Indirect effect | 0.006 | 0.006 | 0.006 |
| Std. Err. | 0.001 | 0.001 | 0.001 |
| z-value | 8.448 | 8.455 | 8.422 |
| p-value | 0.000 | 0.000 | 0.000 |
| CI | 0.005 , 0.008 | 0.005 , 0.008 | 0.005 , 0.008 |
|  | | | |
| Baron and Kenny Approach:  STEP 1 - bmi:salt (X -> M) with B=0.045 and p=0.000  STEP 2 - diabetes:bmi (M -> Y) with B=0.145 and p=0.000  STEP 3 - diabetes:salt (X -> Y) with B=0.004 and p=0.382  As STEP 1, STEP 2 and the Sobel's test above are significant  and STEP 3 is not significant the mediation is complete! | | | |
|  | | | |
| Zhao, Lynch & Chen's approach to testing mediation  STEP 1 - diabetes:salt (X -> Y) with B=0.004 and p=0.382  As the Monte Carlo test above is significant and STEP 1 is not  significant you have indirect-only mediation (full mediation)! | | | |
|  | | | |
| RIT = (Indirect effect / Total effect)  (0.006 / 0.011) = 0.596  Meaning that about 60 % of the effect of salt  on diabetes is mediated by bmi! | | | |
|  | | | |
| RID = (Indirect effect / Direct effect)  (0.006 / 0.004) = 1.474  That is, the mediated effect is about 1.5 times as  large as the direct effect of salt on diabetes! | | | |

Table S5: Mediation analysis of waist circumference on the salt 🡺 diabetes association

| Estimates | Delta | Sobel | Monte Carlo |
| --- | --- | --- | --- |
| Indirect effect | 0.009 | 0.009 | 0.009 |
| Std. Err. | 0.001 | 0.001 | 0.001 |
| z-value | 9.842 | 9.854 | 9.815 |
| p-value | 0.000 | 0.000 | 0.000 |
| CI | 0.008 , 0.011 | 0.008 , 0.011 | 0.008 , 0.011 |
|  | | | |
| Baron and Kenny approach to testing mediation  STEP 1 - wc:salt (X -> M) with B=0.051 and p=0.000  STEP 2 - diabetes:wc (M -> Y) with B=0.183 and p=0.000  STEP 3 - diabetes:salt (X -> Y) with B=0.002 and p=0.735  As STEP 1, STEP 2 and the Sobel's test above are significant  and STEP 3 is not significant the mediation is complete! | | | |
|  | | | |
| Zhao, Lynch & Chen's approach to testing mediation  STEP 1 - diabetes:salt (X -> Y) with B=0.002 and p=0.735  As the Monte Carlo test above is significant and STEP 1 is not  significant you have indirect-only mediation (full mediation)! | | | |
|  | | | |
| RIT = (Indirect effect / Total effect)  (0.009 / 0.011) = 0.848  Meaning that about 85 % of the effect of salt  on diabetes is mediated by wc! | | | |
|  | | | |
| RID = (Indirect effect / Direct effect)  (0.009 / 0.002) = 5.595  That is, the mediated effect is about 5.6 times as  large as the direct effect of salt on diabetes! | | | |
